# VKORC1 sequence variants associated with resistance to anticoagulant rodenticides in Irish populations of *Rattus norvegicus* and *Mus musculus domesticus*

**DOI:** 10.1038/s41598-018-22815-7

**Published:** 2018-03-14

**Authors:** Jean Mooney, Mark R. Lynch, Colin V. Prescott, Tracy Clegg, Michael Loughlin, Bernard Hannon, Colm Moore, Richard Faulkner

**Affiliations:** 1 Molecular Virology Laboratory, Department of Agriculture, Food and the Marine Laboratories, Backweston Campus, Celbridge, Co Kildare Ireland; 2 Chairman, Campaign for Responsible Rodenticide Use Ireland CLG, c/o Glendine, 36 Ludford Drive, Dundrum, Dublin 16 Ireland; 3 School of Animal and Microbial Sciences, The University of Reading, Whiteknights, Reading RG6 6AJ United Kingdom; 4 Centre for Veterinary Epidemiology and Risk Analysis, School of Veterinary Medicine, University College Dublin, Belfield, Ireland; 5 Emel Consulting, Roundwood, Co Wicklow Ireland; 6 Ecolab Ireland, Forest Park, Mullingar Industrial Estate, Mullingar, Co Westmeath Ireland; 7 Rentokil Initial Ltd., Hazel House, Millennium Park, Naas, Co Kildare Ireland

## Abstract

While resistance to anticoagulant rodenticides is known to occur in many European populations of Norway rat and house mouse, to-date no data is available on the occurrence in Ireland of such resistance. No genetic evidence for the occurrence of resistance was found in 65 Norway rat samples analysed, indicative of an absence, or low prevalence, of resistance in rats in at least the Eastern region of the island of Ireland. The presence of two of the most commonly found amino acid substitutions Leu128Ser and Tyr139Cys associated with house mouse resistance to anticoagulant rodenticides was confirmed. The occurrence of two such mutations is indicative of the occurrence of resistance to anticoagulant rodenticides in house mice in the Eastern region of the island of Ireland.

## Introduction

Following their introduction in the 1950s, warfarin and the structurally similar anticoagulant rodenticides (*e.g*. chlorophacinone and coumatetralyl), resulted in radical changes in rodent pest management practice. The newly introduced rodenticides had delayed action, with the result that mortality followed days or weeks after initiation of treatment, an effect that rendered them especially suitable for use to control Norway rats, also known as brown rats (*Rattus norvegicus*), a species that displays neophobic reactions. Reflecting the higher tolerance of house mice (*Mus musculus*) to such compounds, the use of warfarin and other first generation anticoagulants for their control was frequently unsatisfactory^1^.

Following the identification in 1958 of warfarin resistant rat populations in Scotland^2^, such resistance was subsequently confirmed at locations in Wales, in England^3^, in Denmark^4^, in Germany^5^, in Belgium^6^ and is now considered widespread. The first reports of warfarin-resistant mice populations were published in the early 1960s^7^.

As a consequence of increasing concerns generated by the identification of warfarin resistant rodent populations, industry developed the more potent anticoagulant rodenticides bromadiolone, brodifacoum, difenacoum, difethialone and flocoumafen, known as second generation anticoagulant rodenticides. As in the case of the first-generation compounds, these reflect the 4-hydroxycoumarin structure, but display increased lipophilicity that results in their having longer half-lives^8^.

Anticoagulant rodenticides being vitamin K antagonists, block Vitamin K metabolism in the liver by preventing the enzyme vitamin K epoxide reductase (VKOR) from reducing vitamin K epoxide to vitamin K. Vitamin K, an essential co-factor for the activation of a number of vitamin K-dependant coagulation factors, plays an important role in blood coagulation^9^. As a consequence of anticoagulant binding with VKOR, a deficiency of vitamin K and coagulation factors occur, resulting in spontaneous bleeding and eventual death.

In populations of resistant rats, VKOR is slightly changed thus preventing correct binding with the rodenticide which then fails to work^10^, a mechanism based on single nucleotide polymorphisms (SNPs) in the VKORC1 gene that codes for the VKOR enzyme^11^. The VKOR1 gene which has 6126 base pairs and three exons, codes for the VKOR1 protein that contains 163 amino acids^12^. Specific amino acid substitutions in VKORC1 have been shown to confer resistance to anticoagulant rodenticides and are considered to be a prerequisite for the development of rodenticide-resistant populations of rats and mice. Independent mutation events appear to have caused the emergence of resistant populations in different areas, affecting certain amino acid positions of the VKORC1 protein^13,14^. It has been suggested that at least seven independent polymorphisms occur in the VKORC1 gene in Norway rats that provide the genetic basis for anticoagulant resistance in that species, while at least two occur in house mice, although the mutations identified do not explain all aspects of resistance that have been reported^13^.

Scientists in Denmark, Germany and the UK that monitored the evolution and distribution of resistance^3,4,15^, observed that resistance expanded geographically and progressed from warfarin to the more potent active substances bromadiolone and difenacoum. Resistance to brodifacoum has also been reported^16^. Resistance to different anticoagulant rodenticides is known as cross-resistance and evolves from first- to second-generation anticoagulants^5^. As a consequence, resistance to second-generation anticoagulant rodenticides will always be accompanied by resistance to first-generation compounds.

While there have been anecdotal reports of the occurrence of resistance in rodent pest populations in Ireland, to-date there has been no scientific investigation of the matter. The current study was intended to establish if genetic evidence of resistance to anticoagulant rodenticides is present in rodent pest populations on the island of Ireland and if possible to establish the geographical distribution of such populations.

## Materials and Methods

**Figure 1:**
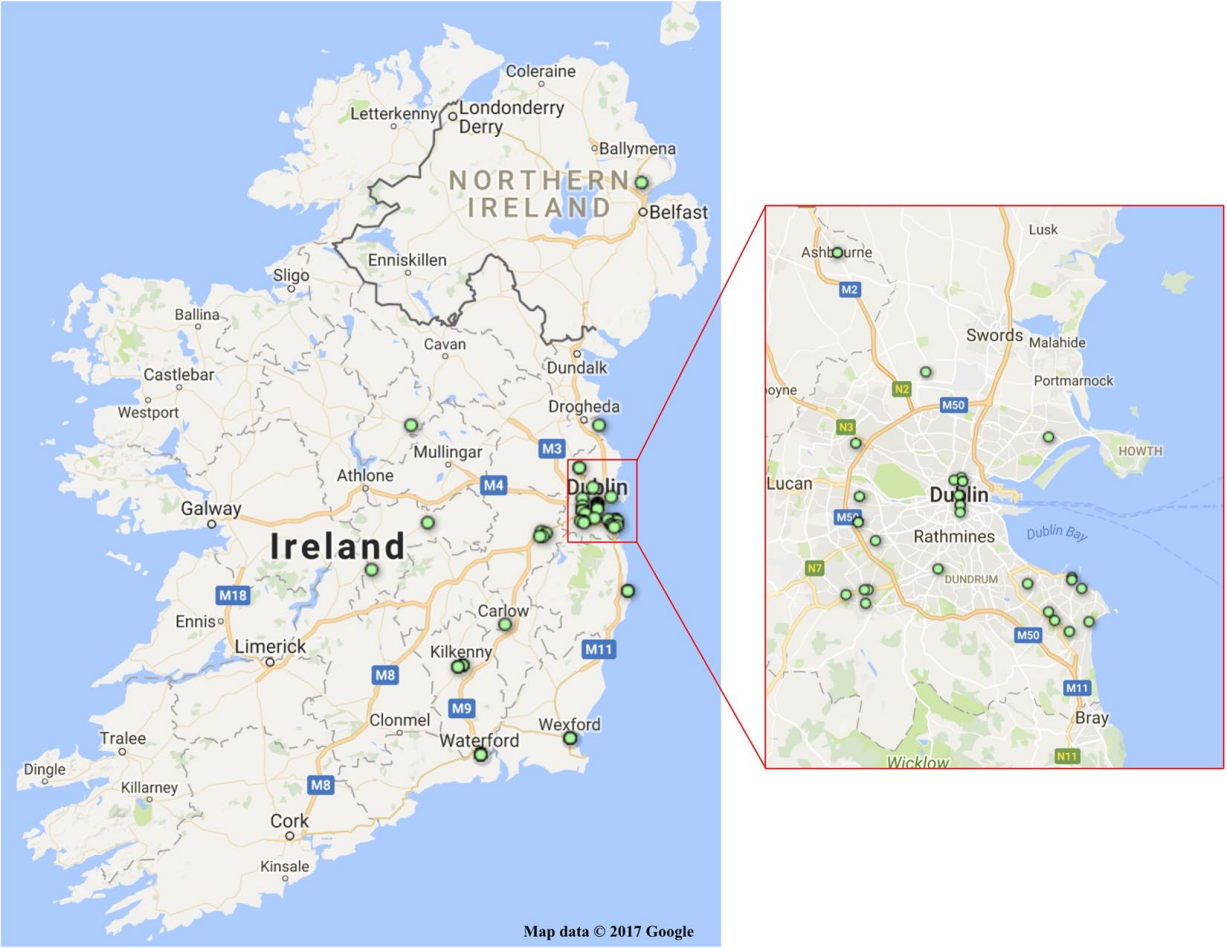
Distribution of Norway Rat Sample Sites. More than one sample was taken at some locations: **Green** indicators show the locations of sampling some 65 rats, none of which had mutations associated with resistance to anticoagulant rodenticides.

**Figure 2:**
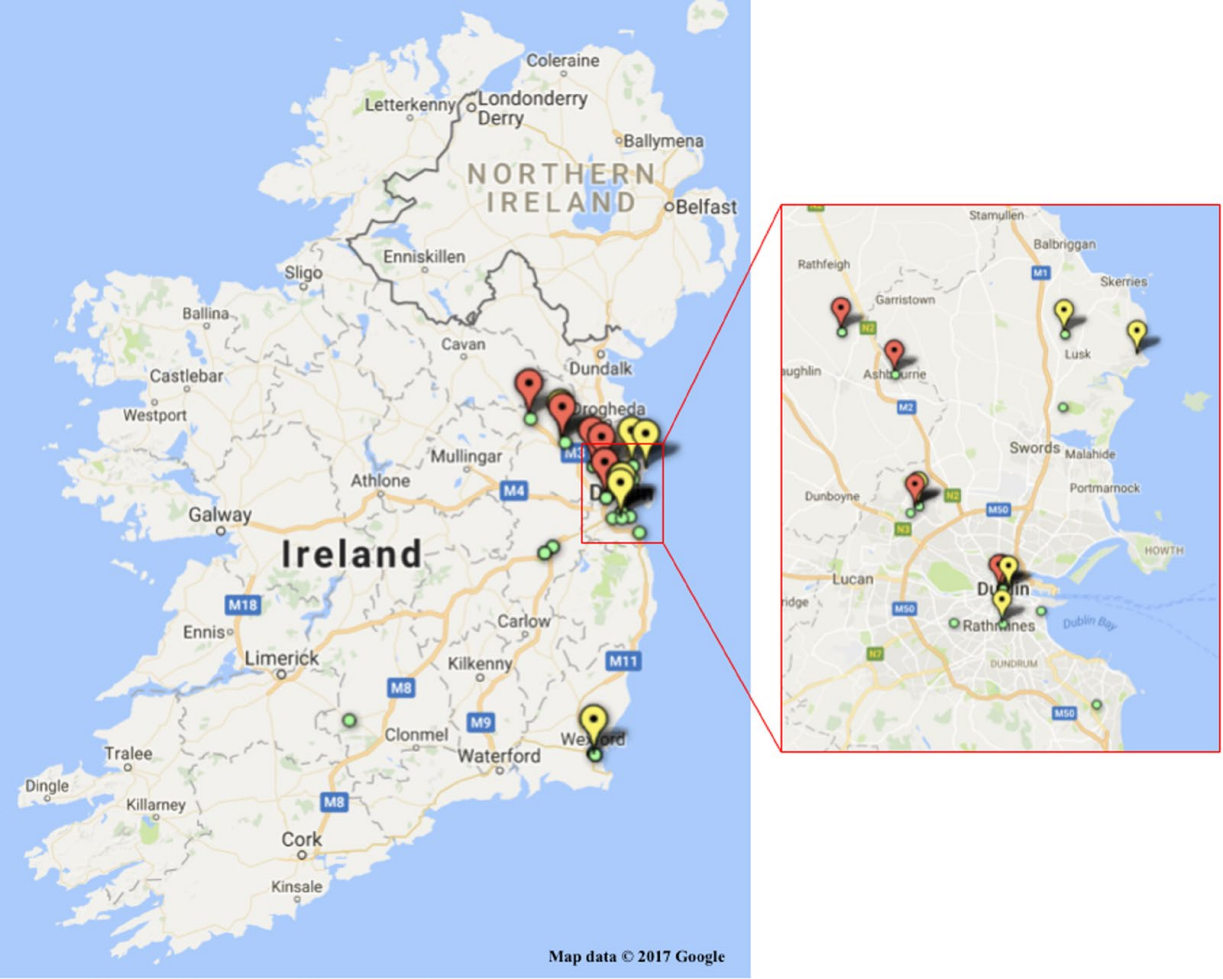
Distribution of House Mouse Sample Sites. More than one sample was taken at some locations: **Yellow** indicators show the locations of sampling 7 mice with a mutation on Exon 3, Codon 128, homozygous Leu128ser L128s, a mutation associated with resistance to warfarin and in some cases to bromadiolone and difenacoum. **Red** indicators show the locations of sampling 7 mice with a mutation on Exon 3, Codon 139, homozygous Tyr139Cys Y139c, a mutation indicative of a high degree of resistance to warfarin and bromadiolone. **Green** indicators show the locations of sampling 28 mice that had heterozygous mutations associated with resistance to anticoagulant rodenticides and 8 that did not have any mutations.

Whole carcases or tail segments of 65 Norway rats and tails of 50 house mice taken between October 2015 and February 2017, were provided by professional pest control officers working in the eastern region of the island of Ireland. The sites from which the samples were provided were distributed throughout the region (Figs 1 and 2). The number of samples provided was considerably less than had been planned and resulted in the project being terminated following the sampling of the eastern region of Ireland. Nevertheless as rodent pests are commensal and as the population of the counties in which sampling took place contain approximately 51% of the population of Ireland in the case of Norway rats and 43% in the case of house mice^17^, the areas sampled provide a satisfactory representation of a large part of the island of Ireland.

On receipt in the laboratory, samples were logged in and stored at −80 °C pending analysis.

Rat tail tissue was excised from tail bone using a scalpel and a fragment of tissue was shaved from the internal membrane of the tail. Mice tails were finely chopped with a scalpel. The tissue fragments were added to 800 µl MagNa Pure Total Nucleic Acid Isolation Kit lysis buffer (Roche Diagnostics Cat No. 03038505001) and 20 µL Proteinase K @ 20 mg/mL (Sigma Cat No. P2308) and digested at 55 °C for 8–24 hours. Total Nucleic acid was extracted from the supernatant on a MagNA Pure LC automated extraction instrument using MagNa Pure Total Nucleic Acid Isolation Kit (Roche Diagnostics Cat No. 03038505001) in accordance with the manufacturer’s instructions. The quality of the extracted nucleic acid was evaluated by testing all extracts for the reference gene ß-actin.

The three exons of the rat VKORC1 gene were amplified by PCR using primers listed in Table 1. The primer sequences for the mouse VKORC1 exons were received from S. Rost, Institut für Humangenetik, Würzburg and were referenced in^14^. PCR reactions on the extracted rat samples were performed with Accustart II PCR ToughMix (Quanta Biosciences Cat. No. 95142-800) at 94 °C for 3 min followed by 40 cycles of 30 sec at 94 °C, 30 sec at 60 °C and 1 min at 72 °C and a final 10 minutes at 72 °C. For the mouse samples the annealing temperature was reduced to 57 °C. The PCR products were cleaned with Illustra ExProStar 1-step Enzymatic PCR and Sequence Reaction Clean-up Kit (GE Healthcare Life Sciences Cat No. US77720), according to the manufacturer’s instructions. The treated extracts were quantified by Qubit prior to sequencing.

**Table 1:** Primer sequences for the PCR amplification of the three exons of the VKORC1 gene in rat samples.

Primer	Sequence 5′-3′	Accession No. and Co-ordinates	Reference*
RE1AF	CTCTTGTGTCTGCGCTGTAC	FQ210919	D. Rymer
RE1R	GCTTTTCATTTCTGCACGCA	HM181985.1 248-229	D. Rymer
RE2CF	GGGTGGCGCTTCTTGCTAA	HM181985.1 942–960	NCBI – Primer BLAST
RE2BR	ACTCCTGCTAAGTGTTCTCCTTG	HM181985.1 1280-1260	NCBI – Primer BLAST
RE3F	TGAGTTCCCTGGTGTCTGTC	HM181985.1 2031-2050	D. Rymer
RE3R	TTTTAGGGACCCACACACGA	HM181985.1 2280-2261	D. Rymer

^*^The rat primer sequences for Exon 1 and 3 were received from D.Rymer, School of Biological Sciences, The University of Reading. Rat exon 2 primers were designed using NCBI primer-BLAST on the Rattus rattus frugivorus Vkorc1 gene HM181985.1.

Sanger sequencing of the extracts, provided by the Molecular Virology Laboratory of the Department of Agriculture, Food and the Marine was conducted by the contract laboratory *Source BioScience*^18^.

## Results and Discussion

Sixty five Norway rat samples (Fig. 1) and fifty house mouse samples (Fig. 2) were successfully extracted and sequenced for all three exons (Tables 2 and 3). The rat sequences were aligned with the NCBI *Rattus norvegicus* VKORC1 reference sequence (NM_203335.2). The mice sequences were aligned with the NCBI *Mus musculus* VKORC1 reference sequence (NM_178600.2). The sequence trace files were visually analysed for the presence of mutations in the three exons when compared with the reference sequences.

**Table 2:** Observed Occurrence by County of Mutations associated with Anticoagulant Resistance in Norway Rats.

County	Sample Numbers Analysed	Mutations found
Antrim	1	0
Carlow	1	0
Dublin	34	0
Kildare	4	0
Kilkenny	11	0
Longford	1	0
Meath	3	0
Offaly	2	0
Waterford	2	0
Wexford	3	0
Wicklow	3	0
Totals	**65**	**0**
95% Confidence Interval		**0.00–5.58%**

**Table 3:** Observed Occurrence by County of Mutations associated with Anticoagulant Resistance in House Mice.

County	Sample Numbers Analysed	Mutation leu128ser L128S (warfarin, bromodialone & difenacoum)		Mutation Tyr139Cys Y139C (warfarin & bromadiolone)	
		Heterozygous	Homozygous	Heterozygous	Homozygous
Dublin	23	9	5	8	3
Kildare	10	4	0	2	0
Meath	7	4	1	5	4
Tipperary	1	1	0	1	0
Wexford	9	4	1	0	0
Totals	**50**	22 (44%)	7 (14%)	16 (32%)	7 (14%)
95% Confidence Intervals		30.0–58.7%	5.8–26.7%	19.5–46.7%	5.8–26.7%

### Rattus novegivcus

There were no polymorphisms (95% CI: 0.00% to 5.58%) in exons 1 and 3 of the rat samples when compared with the *Rattus norvegicus* VKORC1 reference strain (NM_203335.2). A single nucleotide polymorphism (SNP) was observed in codon 82 of exon 2. This point mutation, Ile82Ile, did not result in an amino acid substitution. Fourteen (21.5%, 95% CI: 12.3% to 33.5%) were heterozygous for the point mutation and 7 (11%, 95% CI: 4.4% to 20.9%) were homozygous. The silent mutation Ile82Ile has been detected in *Rattus norvegicus* in England, Europe, Indonesia, Korea, the Azores and North and South America at a high frequency^14^. There is no evidence to date that the silent mutation Il82Il is implicated in anticoagulant resistance, however it has been suggested that the mutation may alter the efficiency of translation of the VKORC1 gene and therefore alter the biological response to anticoagulant exposure^19^.

Rats with the Tyr139Cys, Tyr139Ser and Leu128Gln substitutions in the VKORC1 gene have been found throughout Germany and Switzerland. These mutations are known to confer resistance to anticoagulants or to reduce VKOR activity^13^. Warfarin resistant rats have been shown to be both heterozygous and homozygous for the mutation Tyr139Cys^11^. In Kent the substitution Tyr139Phe was also found in resistant Norway rats at a site treated with bromadiolone bait. All rats tested from the Kent site were found to be homozygous for the mutation^19^.

Mutations associated with resistance to anticoagulant rodenticides have not been detected in Norway rats in this study, indicative of an absence, or low prevalence of genetic evidence of resistance to anticoagulant rodenticides in Norway rats in at least the Eastern region of the island of Ireland.

### Mus musculus domesticus

Leu128Ser and Tyr139Cys are the most frequent amino acid substitutions that have been found to occur in the VKORC1 gene in resistant house mice in the UK and other European countries^20^. A group of linked substitutions Arg12Trp/Ala26Ser/Ala48Thr/Arg61Leu have also been found in mice in Germany and Switzerland and are linked to reduced house mouse susceptibility to anticoagulants^20^. In the UK, house mice resistant to the first-generation anticoagulants are widespread and both Leu128Ser and Tyr139Cys mutations have been found in the VKORC1 gene of resistant mice^13^. In France a mutation Trp59Gly in exon 2 of the VKORC1 gene was found to be associated with warfarin resistant mice^21^.

Of the 50 mice samples tested in this study eight (16%, 95% CI: 7.2% to 29.1%) showed no sequence variation in the VKORC1 gene in comparison with the *Mus musculus* VKORC1 reference sequence (NM_178600.2). In the remaining 84% (95% CI: 70.9% to 92.8%), one or both of the two point mutations Leu128Ser and Tyr139Cys were detected. There was no evidence of the linked substitutions found in mice in Germany and Switzerland or of the substitution Trp59Gly found in mice in France. Twenty two mice tested (44%, 95% CI: 30.0% to 58.7%) were heterozygous for the point mutation Leu128Ser and 7 (14%, 95% CI: 5.8% to 26.7%) were homozygous (Table 3). Sixteen mice (32%, 95% CI: 19.5% to 46.7%) were heterozygous for Tyr139Cys with seven (14%, 95% CI: 5.8% to 26.7%) being homozygous. None of the mice tested were homozygous for both Leu128Ser and Tyr139Cys mutations.

The occurrence of two mutations associated with resistance to anticoagulants rodenticides is indicative of the occurrence of resistance to anticoagulant rodenticides in house mice in at least the Eastern region of the island of Ireland. In similar sized European studies resistance was present in >70–80% of tested mice populations^22,23^. This compares well with the values obtained in this study, in which 78% (95% CI: 56.3% to 92.5%) of mice from the Dublin region had mutations indicative of resistance (based on n = 23 mice), while for the Eastern region of Ireland the value was found to be 84% (95% CI: 70.9% to 92.8%, based on n = 50 mice).

## Conclusions

Although the number of samples available for analysis (65) was less than planned, no genetic evidence was found of the occurrence and distribution of mutations associated with Norway rat resistance to anticoagulant rodenticides in the Eastern region of Ireland. Having regard to comparable European studies with similar sample numbers, the absence of genetic evidence of resistance in the 65 samples taken in the Eastern part of Ireland, suggests that there is a high probability that these are true negative findings or that the prevalence is very low.

The data generated confirmed the widespread presence of mutations that result in the two most commonly found amino acid substitutions, Leu128Ser and Tyr139Cys, associated with house mice resistance to anticoagulant rodenticides in the Eastern region of Ireland, but these were not present in samples from all counties in which samples were collected (see Supplementary Information Table). Of the 6 mutations for which screening was conducted (Table 1), only Leu128Ser and Tyr139Cys were detected.

No conclusions can be drawn concerning the occurrence of those mutations in the rest of the island of Ireland. Biological testing would be required to establish the extent to which those mutations have conferred field resistance to the house mouse populations concerned, however it is highly likely that such field resistance is present in house mouse populations in at least the Eastern Region of Ireland.

The probability remains that field resistance to anticoagulant rodenticides is present in populations of house mice in at least the Eastern region of Ireland. The use of anticoagulants in attempts to control rodent pests that have developed resistance to them unnecessarily exposes wildlife to poisoning with rodenticides.

Rodent pests are vectors or carry vectors of many serious human and animal diseases, cause significance spoilage, losses of food and feed as well as damage to buildings and infrastructure. There are around 200 rodenticide products authorised for use in Ireland^24^, and in excess of 60 companies provide pest control services in Ireland^25^. There is a clear need for further research to establish the extent and distribution of anticoagulant resistance in populations of rodent pests on the island of Ireland. Given the challenges identified in collecting sufficient numbers of samples from farms, industrial and urban areas on a goodwill basis, it is recommended that any future study be funded such that relevant voluntary organisation personnel can be trained in both sample collection and handling under the supervision of a PhD student selected to lead the project. Since a key goal of acquiring such information is to allow assessment of the extent of resistant populations of rodents and consequently of the extent of unnecessary exposure of non-target wildlife to rodenticides though both primary and secondary poisoning, the preferred approach is to utilise samples acquired along rural to urban gradients as per the initial strategy utilised in this study.

## Electronic supplementary material

Dataset 1
