# Supplementary material for: VKORC1 sequence variants associated with resistance to anticoagulant rodenticides in Irish populations of Rattus norvegicus and Mus musculus domesticus
Source: Sci Rep. 2018 Mar 14;8:4535. doi: 10.1038/s41598-018-22815-7 (PMC5852000; doi:10.1038/s41598-018-22815-7)
Supplement: Supplementary file 1 — Dataset 1 [file 41598_2018_22815_MOESM1_ESM.doc]

**To date on sequencing samples 29/8/17**

**Mouse Samples** – 50 samples sequenced in all three exons. 10 samples not suitable for testing

No mutations on Exon 1 or Exon 2. Tyr139CYs prevails in Denmark, Germany, parts of Azores, France, Holland and Hungary. The Tyr139cys and Leu128ser mutations in mice are also widespread in the UK and the house mice carrying the homozygous Y139C sequence variant were found to be highly resistant to warfarin and bromadiolone. House mice carrying the homozygous l128S are resistant to warfarin and some are resistant to bromodiolone and difenacoum.

**Exon 3:**

Codon 128 22/50 = 44% TTA/TCA heterozygous

21/50 = 42% TTA homozygous (wild type)

7/50 = 14% TCA homozygous - leu128ser L128S

Codon 139 16/50 = 32% TAT/TGT heterozygous

27/50 = 54% TAT homozygous (wild type)

7/50 = 14% TGT homozygous Tyr139Cys Y139C

Codon 128 + 139 12/50 = 24% TTA/TCA + TAT

9/50 = 18% TTA/TCA + TAT/TGT

8/50 = 16% TTA + TAT (wild type)

6/50 = 12% TTA + TAT/TGT

7/50 = 14% TTA + TGT

1/50 = 2%% TCA + TAT/TGT

6/50 = 12% TCA + TAT

**Rat Samples** - 65 samples sequenced in all three exons

No mutations on RE1 or RE3. The base change on codon 82 of Exon 2 does not result in an amino acid change as both ATA and ATT code for Isoleucine.

Exon 2:

Codon 82 7/65 = 11% ATT homozygous

14/65 = 21.5% ATA/ATT heterozygous

44/65 = 67.5% ATA homozygous (wild type)

**Mouse Samples**

|  |  | Sequence | | | | | | | | Area | Premises | Company |
| --- | --- | --- | --- | --- | --- | --- | --- | --- | --- | --- | --- | --- |
| Sample | GV No. | E1  26 | E1  35 | E1  56 | E2  79 | E2  82 | E3  120 | E3  128 | E3  139 |  |  |  |
| Mouse | GV15-036997 | GCA | CGC | TCC | TCC | ATA | CTG | TYA | TAT | Rathomooney, Lusk, Co Dublin | Farm | Ecolab |
| Mouse | GV15-036998 | GCA | CGC | TCC | TCC | ATA | CTG | TYA | TRT | Rathomooney, Lusk, Co Dublin | Farm | Ecolab |
| Mouse | GV15-036999 | GCA | CGC | TCC | TCC | ATA | CTG | TCA | TRT | Rathomooney, Lusk, Co Dublin | Farm | Ecolab |
| Mouse | GV15-037000 | GCA | CGC | TCC | TCC | ATA | CTG | TTA | TAT | Rathomooney, Lusk, Co Dublin | Farm | Ecolab |
| Mouse | GV15-037166 | GCA | CGC | TCC | TCC | ATA | CTG | TTA | TRT | Dublin | No info | No info |
| Mouse | GV15-037167 | GCA | CGC | TCC | TCC | ATA | CTG | TYA | TRT | Dublin | No info | No info |
| Mouse | GV15-037168 | GCA | CGC | TCC | TCC | ATA | CTG | TTA | TGT | Dublin | No info | No info |
| Mouse | GV15-037169 | GCA | CGC | TCC | TCC | ATA | CTG | TTA | TAT | Dublin | No info | No info |
| Mouse | GV15-037170 | GCA | CGC | TCC | TCC | ATA | CTG | TYA | TAT | Dublin | No info | No info |
| Mouse | GV15-037230 | GCA | CGC | TCC | TCC | ATA | CTG | TYA | TRT | Tougher Business Park, Kildare | Bakery | Cannon |
| Mouse | GV15-037238 | GCA | CGC | TCC | TCC | ATA | CTG | TYA | TAT | Drimnagh Road Retail Park, Dublin | No info | Cannon |
| Mouse | GV15-037239 | GCA | CGC | TCC | TCC | ATA | CTG | TYA | TAT | Tougher Business Park, Kildare | Business Park | Cannon |
| Mouse | GV15-037258 | GCA | CGC | TCC | TCC | ATA | CTG | TCA | TAT | Dublin | No info | No info |
| Mouse | GV15-039025 | GCA | CGC | TCC | TCC | ATA | CTG | TTA | TGT | Dublin | ILAC Centre | Ecolab |
| Mouse | GV15-039050 | GCA | CGC | TCC | TCC | ATA | CTG | TCA | TAT | Rush, Co Dublin | Tesco | Ecolab |
| Mouse | GV15-041745 | GCA | CGC | TCC | TCC | ATA | CTG | TYA | TAT | Largo Foods, Kilbreen, Ashbourne, Co Meath | Meat Products Factory | Ecolab |
| Mouse | GV15-041746 | GCA | CGC | TCC | TCC | ATA | CTG | TTA | TRT | Largo Foods, Kilbreen, Ashbourne, Co Meath | Meat Products Factory | Ecolab |
| Mouse | GV15-041747 | GCA | CGC | TCC | TCC | ATA | CTG | TYA | TRT | Largo Foods, Kilbreen, Ashbourne, Co Meath | Meat Products Factory | Ecolab |
| Mouse | GV15-041748 | GCA | CGC | TCC | TCC | ATA | CTG | TTA | TGT | Largo Foods, Kilbreen, Ashbourne, Co Meath | Meat Products Factory | Ecolab |
| Mouse | GV15-041749 | GCA | CGC | TCC | TCC | ATA | CTG | TTA | TRT | Largo Foods, Kilbreen, Ashbourne, Co Meath | Meat Products Factory | Ecolab |
| Mouse | GV15-044244 | GCA | CGC | TCC | TCC | ATA | CTG | TTA | TRT | Roslyn Park, St. Margaret’s, Dublin | Food Warehouse | Cannon |
| Mouse | GV15-045330 | GCA | CGC | TCC | TCC | ATA | CTG | TYA | TAT | Clouglands Bakery, Newbridge, Co. Kildare | Bakery | Ecolab |
| Mouse | GV15-045333 | GCA | CGC | TCC | TCC | ATA | CTG | TTA | TRT | Clouglands Bakery, Newbridge, Co. Kildare | Bakery | Ecolab |
| Mouse | GV15-045334 | GCA | CGC | TCC | TCC | ATA | CTG | TYA | TAT | Clouglands Bakery, Newbridge, Co. Kildare | Bakery | Ecolab |
| Mouse | GV16-005427 | GCA | CGC | TCC | TCC | ATA | CTG | TTA | TAT | Tesco, Donabate, Dublin | Tesco | Rentokill |
| Mouse | GV16-005432 | GCA | CGC | TCC | TCC | ATA | CTG | TYA | TAT | Musgrave, Blanchardstown, Dublin | Musgraves | Ecolab |
| Mouse | GV16-005433 | GCA | CGC | TCC | TCC | ATA | CTG | TCA | TAT | Musgrave, Blanchardstown, Dublin | Musgraves | Ecolab |
| Mouse | GV16-005434 | GCA | CGC | TCC | TCC | ATA | CTG | TTA | TRT | Musgrave, Blanchardstown, Dublin | Musgraves | Ecolab |
| Mouse | GV16-005435 | GCA | CGC | TCC | TCC | ATA | CTG | TTA | TGT | Musgrave, Blanchardstown, Dublin | Musgraves | Ecolab |
| Mouse | GV16-005436 | GCA | CGC | TCC | TCC | ATA | CTG | TCA | TAT | Athgaine, Navan, Co Meath | Factory, engineering | Ecolab |
| Mouse | GV16-005437 | GCA | CGC | TCC | TCC | ATA | CTG | TYA | TRT | Arcroyal, Kells, Co Meath | Factory, engineering | Ecolab |
| Mouse | GV16-005438 | GCA | CGC | TCC | TCC | ATA | CTG | TTA | TGT | Arcroyal, Kells, Co Meath | Factory, engineering | Ecolab |
| Mouse | GV16-005450 | GCA | CGC | TCC | TCC | ATA | CTG | TTA | TGT | Ardboyne Hotel, Navan, Co Meath | Ardboyne Hotel | Ecolab |
| Mouse | GV16-005451 | GCA | CGC | TCC | TCC | ATA | CTG | TYA | TRT | Lir, Navan, Co Meath | Factory, Milk Products | Ecolab |
| Mouse | GV16-005453 | GCA | CGC | TCC | TCC | ATA | CTG | TYA | TRT | Alexxion, Blanchardstown, Co Dublin | Factory, engineering | Ecolab |
| Mouse | GV16-008399 | GCA | CGC | TCC | TCC | ATA | CTG | TCA | TAT | Belgrove Sq, Rathmines, Dublin | House | Total pest Control |
| Mouse | GV16-008402 | GCA | CGC | TCC | TCC | ATA | CTG | TTA | TAT | Belgrove Sq, Rathmines, Dublin | House | Total pest Control |
| Mouse | GV16-034518 | GCA | CGC | TCC | TCC | ATA | CTG | TTA | TAT | Granville Tce, DunLaoghaire, Co Dublin | House | Rentokill |
| Mouse | GV16-036269 | GCA | CGC | TCC | TCC | ATA | CTG | TTA | TAT | Glanbia, Wexford | Glanbia | Ecolabs |
| Mouse | GV16-036270 | GCA | CGC | TCC | TCC | ATA | CTG | TYA | TAT | Glanbia, Wexford | Glanbia | Ecolabs |
| Mouse | GV16-036271 | GCA | CGC | TCC | TCC | ATA | CTG | TCA | TAT | Glanbia, Wexford | Glanbia | Ecolabs |
| Mouse | GV16-036272 | GCA | CGC | TCC | TCC | ATA | CTG | TYA | TAT | Glanbia, Wexford | Glanbia | Ecolabs |
| Mouse | GV16-036273 | GCA | CGC | TCC | TCC | ATA | CTG | TTA | TAT | Glanbia, Wexford | Glanbia | Ecolabs |
| Mouse | GV16-036274 | GCA | CGC | TCC | TCC | ATA | CTG | TYA | TAT | Glanbia, Wexford | Glanbia | Ecolabs |
| Mouse | GV16-036276 | GCA | CGC | TCC | TCC | ATA | CTG | TYA | TAT | Glanbia, Wexford | Glanbia | Ecolabs |
| Mouse | GV16-036530a | GCA | CGC | TCC | TCC | ATA | CTG | TYA | TAT | Wexford | Food Premises | Ecolabs |
| Mouse | GV16-036530c | GCA | CGC | TCC | TCC | ATA | CTG | TYA | TRT | Wexford | Food Premises | Ecolabs |
| Mouse | GV16-036786a | GCA | CGC | TCC | TCC | ATA | CTG | TTA | TGT | Ashbourne, Co. Meath | Food Premises | Ecolabs |
| Mouse | GV16-036786b | GCA | CGC | TCC | TCC | ATA | CTG | TTA | TAT | Ashbourne, Co. Meath | Food Premises | Ecolabs |
| Mouse | GV16-038646 | GCA | CGC | TCC | TCC | ATA | CTG | TYA | TRT | Tipperary | Farm | Private, C Gallagher |

50 Mice samples sequenced in all three exons, 10 mice samples not suitable for testing or did not have good quality sequence for all three exons.

| Mouse | GV16-005426 | Poor Sample Quality | Tesco, Donabate, Co Dublin |  | Rentokil |
| --- | --- | --- | --- | --- | --- |
| Mouse | GV16-005428 | Poor Sample Quality | Tesco, Donabate, Co Dublin |  | Rentokil |
| Mouse | GV16-005429 | Poor Sample Quality | Cappagh Hospital, Cappagh, Co Dublin |  | Ecolab |
| Mouse | GV16-005430 | Poor Sample Quality | Cappagh Hospital, Cappagh, Co Dublin |  | Ecolab |
| Mouse | GV16-005452 | Poor Sample Quality | Butterfly Cafe, Kells, Co Meath |  | Ecolab |
| Mouse | GV16-005454 | Poor Sample Quality | Musgraves, Blanchardstown, Co Dublin |  | Ecolab |
| Mouse | GV16-003565 | Poor Sample Quality | Killeavy, Armagh |  | Private |
| Mouse | GV16-003566 | Poor Sample Quality | Killeavy Armagh |  | Private |
| Mouse | GV16-036275 | Poor Sample Quality | Glanbia, Wexford |  | Ecolabs |
| Mouse | GV16-036530b | Poor Sample Quality | Wexford | Food Premises | Ecolabs |

**Rat Samples**

|  |  | Sequence | | | | | | | |  |  |  |
| --- | --- | --- | --- | --- | --- | --- | --- | --- | --- | --- | --- | --- |
| Sample Type | GV No. | E1  26 | E1  35 | E1  56 | E2  79 | E2  82 | E3  120 | E3  128 | E3  139 |  |  |  |
| Rat | GV15-037290 | GCA | CGC | TCC | TCC | ATW | CTG | CTG | TAT | Whitworth Place, Drumcondra, Dublin | House | Cannon |
| Rat | GV15-037323 | GCA | CGC | TCC | TCC | ATA | CTG | CTG | TAT | Mater Hospital, Eccles St, Dublin | Hospital | Cannon |
| Rat | GV15-037830 | GCA | CGC | TCC | TCC | ATA | CTG | CTG | TAT | St Berach’s Place, Dublin | Community Centre | DCC |
| Rat | GV15-040823 | GCA | CGC | TCC | TCC | ATA | CTG | CTG | TAT | C&D Foods, Edgeworthstown, Co. Longford | Meat Processing | Ecolab |
| Rat | GV15-041776 | GCA | CGC | TCC | TCC | ATA | CTG | CTG | TAT | Ashbourne, Co Meath | Largo Foods | Ecolab |
| Rat | GV15-041777 | GCA | CGC | TCC | TCC | ATA | CTG | CTG | TAT | Ashbourne, Co Meath | Largo Foods | Ecolab |
| Rat | GV15-044238 | GCA | CGC | TCC | TCC | ATA | CTG | CTG | TAT | North Earl St., Dublin | Bakery | Ecolab |
| Rat | GV15-044239 | GCA | CGC | TCC | TCC | ATA | CTG | CTG | TAT | North Earl St., Dublin | Bakery | Ecolab |
| Rat | GV15-044245 | GCA | CGC | TCC | TCC | ATA | CTG | CTG | TAT | St. Margaret’s, Co Dublin | Roslyn park | Cannon |
| Rat | GV15-044461 | GCA | CGC | TCC | TCC | ATA | CTG | CTG | TAT | Stephens Green, Dublin |  | Ecolab |
| Rat | GV15-045123 | GCA | CGC | TCC | TCC | ATW | CTG | CTG | TAT | Bettystown, Co Meath | No info | Ecolab |
| Rat | GV15-045337 | GCA | CGC | TCC | TCC | ATW | CTG | CTG | TAT | Raheen Drive, Tallaght, Dublin | House | Ecolab |
| Rat | GV16-002979 | GCA | CGC | TCC | TCC | ATA | CTG | CTG | TAT | Merrywell Industrial Est., Ballymount, Dublin 12 | Irish packaging recycling | Cannon |
| Rat | GV16-004493 | GCA | CGC | TCC | TCC | ATA | CTG | CTG | TAT | Oakcourt Av, Palmerstown, Co. Dublin | House | Cannon |
| Rat | GV16-005405 | GCA | CGC | TCC | TCC | ATT | CTG | CTG | TAT | 123 Portland St., Dublin 1 | House | Rentokil |
| Rat | GV16-005406 | GCA | CGC | TCC | TCC | ATA | CTG | CTG | TAT | 124 Portland St., Dublin 1 | House | Rentokil |
| Rat | GV16-005722 | GCA | CGC | TCC | TCC | ATW | CTG | CTG | TAT | Cabinteely Av, Dublin | House | Total pest Control |
| Rat | GV16-005725 | GCA | CGC | TCC | TCC | ATT | CTG | CTG | TAT | Suffolk St, Dublin | Superdry | Ecolab |
| Rat | GV16-008396 | GCA | CGC | TCC | TCC | ATA | CTG | CTG | TAT | Churchview, Ballybrack, Co Dublin | House, Flats |  |
| Rat | GV16-009597 | GCA | CGC | TCC | TCC | ATA | CTG | CTG | TAT | Cloister Way, Blackrock, Co Dublin | House, Flats | Total pest Control |
| Rat | GV16-009605 | GCA | CGC | TCC | TCC | ATA | CTG | CTG | TAT | Clonheen Rd., Deansgrange, Dublin | House | Total pest Control |
| Rat | GV16-009606 | GCA | CGC | TCC | TCC | ATA | CTG | CTG | TAT | Dublin | House, Flats | Total pest Control |
| Rat | GV16-33014 | GCA | CGC | TCC | TCC | ATA | CTG | CTG | TAT | Kilkenny | Offices | Pestkill |
| Rat | GV16-34509 | GCA | CGC | TCC | TCC | ATW | CTG | CTG | TAT | Jobstown, Tallaght, Dublin | House | Rentokil |
| Rat | GV16-34512 | GCA | CGC | TCC | TCC | ATW | CTG | CTG | TAT | Marine Rd., DunLaoghaire, Co Dublin | Food Premises | Rentokil |
| Rat | GV16-34513 | GCA | CGC | TCC | TCC | ATA | CTG | CTG | TAT | Marine Rd., DunLaoghaire, Co Dublin | Food Premises | Rentokil |
| Rat | GV16-34631 | GCA | CGC | TCC | TCC | ATA | CTG | CTG | TAT | Bellview Port, Ferrybank, Belview, Co Kilkenny | Animal Feed | Robinson pest Control |
| Rat | GV16-34639 | GCA | CGC | TCC | TCC | ATW | CTG | CTG | TAT | Anne St., Ferrybank South, Wexford | Government building | Cannon |
| Rat | GV16-34656 | GCA | CGC | TCC | TCC | ATA | CTG | CTG | TAT | Carlow | Restaurant | Ecolab |
| Rat | GV16-34911 | GCA | CGC | TCC | TCC | ATW | CTG | CTG | TAT | DunLaoghaire, Co Dublin | House, Flats | Cannon |
| Rat | GV16-35479 | GCA | CGC | TCC | TCC | ATA | CTG | CTG | TAT | Wicklow | Food Premises | Ecolab |
| Rat | GV16-35600 | GCA | CGC | TCC | TCC | ATA | CTG | CTG | TAT | Waterford | Chicken Farm | Ecolab |
| Rat | GV16-35649 | GCA | CGC | TCC | TCC | ATA | CTG | CTG | TAT | Kilkenny |  | Ecolab |
| Rat | GV16-35807 | GCA | CGC | TCC | TCC | ATW | CTG | CTG | TAT | Kilkenny | Food Premises | Pestkill |
| Rat | GV16-35810 | GCA | CGC | TCC | TCC | ATT | CTG | CTG | TAT | Waterford | Meat Processing | Ecolab |
| Rat | GV16-36363 | GCA | CGC | TCC | TCC | ATA | CTG | CTG | TAT | Wexford | Manufacturing Plant | Ecolab |
| Rat | GV16-36364 | GCA | CGC | TCC | TCC | ATA | CTG | CTG | TAT | Wexford | Manufacturing Plant | Ecolab |
| Rat | GV16-36513 | GCA | CGC | TCC | TCC | ATW | CTG | CTG | TAT | Wicklow | IDA –Food Processing | Ecolab |
| Rat | GV16-36525 | GCA | CGC | TCC | TCC | ATW | CTG | CTG | TAT | Wicklow | Factory | Cannon |
| Rat | GV16-36778 | GCA | CGC | TCC | TCC | ATA | CTG | CTG | TAT | Drakelands, Co. Kilkenny | Grain Store | Pestkill |
| Rat | GV16-36779 | GCA | CGC | TCC | TCC | ATA | CTG | CTG | TAT | Drakelands, Co. Kilkenny | Grain Store | Pestkill |
| Rat | GV16-36780 | GCA | CGC | TCC | TCC | ATT | CTG | CTG | TAT | Drakelands, Co. Kilkenny | Grain Store | Pestkill |
| Rat | GV16-36781 | GCA | CGC | TCC | TCC | ATW | CTG | CTG | TAT | Drakelands, Co. Kilkenny | Grain Store | Pestkill |
| Rat | GV16-37447 | GCA | CGC | TCC | TCC | ATA | CTG | CTG | TAT | Millenium Park, Naas, Co Kildare | Food Premises | Rentokil |
| Rat | GV16-37450 | GCA | CGC | TCC | TCC | ATA | CTG | CTG | TAT | Old Bawn, Tallaght, Co Dublin | Public House | Rentokil |
| Rat | GV16-37858 | GCA | CGC | TCC | TCC | ATT | CTG | CTG | TAT | Tallaght By-pass, Tallaght, Co. Dublin | Garage | Rentokil |
| Rat | GV16_38893 | GCA | CGC | TCC | TCC | ATA | CTG | CTG | TAT | Glasthule Rd, Sandycove, Co. Dublin | House | Total Pest Control |
| Rat | GV16_39051 | GCA | CGC | TCC | TCC | ATA | CTG | CTG | TAT | Ballyclover, Co. Antrim | house | Robinson pest Control |
| Rat | GV16_39477 | GCA | CGC | TCC | TCC | ATA | CTG | CTG | TAT | Tullamore, Co Offaly | KMK recycle Scrap metal | Ecolabs |
| Rat | GV16_39479 | GCA | CGC | TCC | TCC | ATA | CTG | CTG | TAT | Rathfarnham, Dublin | House | Cannon |
| Rat | GV16_39569 | GCA | CGC | TCC | TCC | ATW | CTG | CTG | TAT | Suffolk St., Dublin | Restaurant | Total Pest Control |
| Rat | GV16_39594 | GCA | CGC | TCC | TCC | ATT | CTG | CTG | TAT | Killiney, Co. Dublin | House | Total Pest Control |
| Rat | GV17_598 | GCA | CGC | TCC | TCC | ATA | CTG | CTG | TAT | Park West, New Nangor Rd, Dublin 12 | Industry | Rentokill |
| Rat | GV17_599 | GCA | CGC | TCC | TCC | ATA | CTG | CTG | TAT | Park West, New Nangor Rd, Dublin 12 | Industry | Rentokill |
| Rat | GV17_600 | GCA | CGC | TCC | TCC | ATA | CTG | CTG | TAT | Park West, New Nangor Rd, Dublin 12 | Industry | Rentokill |
| Rat | GV17_634 | GCA | CGC | TCC | TCC | ATA | CTG | CTG | TAT | Park West, New Nangor Rd, Dublin 12 | Hotel | Rentokill |
| Rat | GV17_635 | GCA | CGC | TCC | TCC | ATA | CTG | CTG | TAT | Park West, New Nangor Rd, Dublin 12 | Hotel | Rentokill |
| Rat | GV17_643 | GCA | CGC | TCC | TCC | ATW | CTG | CTG | TAT | Blanchardstown, Co Dublin | House | Rentokill |
| Rat | GV17_1105 | GCA | CGC | TCC | TCC | ATA | CTG | CTG | TAT | Kilkenny | House | EMEL Consulting |
| Rat | GV17_1106 | GCA | CGC | TCC | TCC | ATA | CTG | CTG | TAT | Kilkenny | House | EMEL Consulting |
| Rat | GV17_1107 | GCA | CGC | TCC | TCC | ATA | CTG | CTG | TAT | Kilkenny | House | EMEL Consulting |
| Rat | GV17_3012 | GCA | CGC | TCC | TCC | ATA | CTG | CTG | TAT | Naas, Co Kildare | Industrial Est. | Rentokill |
| Rat | GV17_3015 | GCA | CGC | TCC | TCC | ATA | CTG | CTG | TAT | Jigginstown Park, Naas, Co Kildare | House | Rentokill |
| Rat | GV17_5105 | GCA | CGC | TCC | TCC | ATA | CTG | CTG | TAT | Fishery Lane, Naas, Co Kildare | House | Rentokill |
| Rat | GV17_7867 | GCA | CGC | TCC | TCC | ATT | CTG | CTG | TAT | Crinkill, Birr, Co Offaly | Grant Engineering | Ecolabs |

65 rats sequenced

| Rat | GV16-021992 | Poor Sample Quality | Rathfarnham, Dublin |  | Complete Pest Control |
| --- | --- | --- | --- | --- | --- |
| Rat | GV16-37436 | No seq for E2 | Glenageary, Co Dublin | House | Rentokil |
| Rat | GV16-33832 | No seq for E3 | Killiney, Co Dublin | House | Cannon |

68 rats in total received.

**VKORC1 sequence variants associated with resistance to anticoagulant rodenticides in Irish populations of *Rattus norvegicus* and *Mus musculus domesticus***

Jean Mooney^*^, Mark R Lynch^#^, Colin V Prescott^‡^, Tracy Clegg^∆^, Michael Loughlin^∫^, Bernard Hannon^a^, Colm Moore^b^ and Richard Faulkner^b^

^*^ Molecular Virology Laboratory, Department of Agriculture, Food and the Marine Laboratories, Backweston Campus, Celbridge, Co Kildare, Ireland

^#^ Chairman, Campaign for Responsible Rodenticide Use Ireland, [info@lynchconsulting.ie](mailto:info@lynchconsulting.ie)

^‡^ School of Animal and Microbial Sciences, The University of Reading, Whiteknights, Reading RG6 6AJ, United Kingdom

^∆^ Centre for Veterinary Epidemiology and Risk Analysis, School of Veterinary Medicine, University College Dublin, Belfield, Ireland

^∫^ Emel Consulting, Roundwood, Co Wicklow, Ireland

^a^ Ecolab Ireland, Forest Park, Mullingar Industrial Estate, Mullingar, Co Westmeath, Ireland

^b^ Rentokil Initial Ltd., Hazel House, Millennium Park, Naas, Co Kildare, Ireland
